# A practical prioritization strategy for vegetation‐focused restoration of anthropogenic wastelands

**DOI:** 10.1002/ajb2.70273

**Published:** 2026-10-04

**Authors:** Erika Stewart, Joshua B. Grinath, Kathryn G. Turner

**Affiliations:** ^1^ Idaho State University 921 S. 8th Ave Pocatello 83209 USA Idaho

**Keywords:** adaptive management, anthropogenic disturbance, classical management, degraded ecosystems, ecosystem recovery, habitat proximity, landscape fragmentation, propagule pressure, restoration ecology, spatial scale

Restoration ecology is increasingly discussed by the scientific community and policy makers, with the UN declaring this the decade on ecosystem restoration (United Nations, 2025). Restoration is often defined as assisting in the recovery of a degraded or damaged ecosystem (Hobbs, 2016) and aims to restore ecosystem function and health (Klaus and Kiehl, 2021). While what defines a degraded ecosystem is debated, it has been argued that severely degraded ecosystems are easily recognized by the loss of biodiversity, lack of vegetation and the ability to support other organisms, or decline in ecosystem services such as nutrient cycling (Hobbs, 2016). At present, few ecosystems are now considered original, intact, or unaffected by human influence (Seastedt et al., 2008), and restoration activities are more widespread than ever before. Restoration often incurs high costs for establishment and maintenance, and to combat invasive species (Bodin et al., 2022; Fantle‐Lepczyk et al., 2022), and restoration projects remain heavily underfunded, with a global gap of $300 billion estimated in annual funding (Bodin et al., 2022). With such limited resources, it is imperative that efforts are focused on restoring areas with high likelihood of success; however, restoration efforts are frequently unsuccessful, especially in areas considered anthropogenic wastelands (Richardson et al., 2010).

Here, we define *anthropogenic wastelands* as severely degraded ecosystems where vegetation and soils have been heavily affected by anthropogenic inputs (e.g., herbicide, waste, grazing, invasive species) and altogether lack recognizable characteristics of ecosystem functioning (Richardson et al., 2010; Ohsowski et al., 2012). In this context, functioning arid ecosystems (drylands/deserts) are clearly not wastelands in need of restoration (Hoover et al., 2020). Of the research to date, most approaches to restore anthropogenic wastelands have failed when attempted on the constrained budgets available (Richardson et al., 2010). Moreover, there is little literature on the restoration of anthropogenic wasteland sites. A literature search in Web of Science (website https://www.webofscience.com) returns fewer than 15 publications/year before 2018, and 15–30 publications/year from 2019 to present (Clarivate, 2026). This is likely due in part to the implicit assumption that wastelands require significantly more management inputs than less‐degraded ecosystems to achieve *any* restoration goals (Hobbs and Norton, 1996). Altogether, these factors necessitate that land managers develop practical prioritization strategies and approaches for anthropogenic wasteland restoration.

In anthropogenic wastelands, organisms have been subject to drastic changes that may be irreversible, historic conditions cannot be maintained, and it is unknown if baseline conditions can support restoration efforts (King and Hobbs, 2006; Ohsowski et al., 2012). Restoration attempts typically include physical, chemical, and biological remediation strategies, often at large scales, but with little consideration of multiple restoration goals (Gawecka and Bascompte, 2023). Management goals may shift from restoration of historic conditions to the establishment of a novel ecosystem (Richardson et al., 2010; Lockwood et al., 2013), or an intermediate, hybrid ecosystem (Klaus and Kiehl, 2021). Classical restoration strategies focus on the removal of invasive species and restoration of native vegetation, assuming that this will eventually restore functional biodiversity and ecosystem services (Hobbs, 2016). Alternatively, managers may employ adaptive restoration strategies that use multiple methods, including encouraging species interactions, to meet multiple goals capable of multiple functional endpoints, and may include the creation of a novel ecosystem (Hobbs, 2016).

To achieve successful restoration of anthropogenic wastelands, practitioners may need to test, then adopt adaptive management strategies where restored communities are capable of differential responses to disturbance through time (Choi, 2007; Ohsowski et al., 2012). Of the few published studies that have successfully achieved restoration goals at anthropogenic wasteland sites, some advocate for site specific strategies, such as using plant species from biophysically analogous ecosystems for restoration (Richardson et al., 2010), while others advocate for maximizing diversity in the face of uncertainty (Seastedt et al., 2008). At present, there are few frameworks for restoration practitioners to rely upon for guidance in attempting restoration of this kind.

Gawecka and Bascompte (2023) propose a framework for optimizing restoration that highlights ecological interactions using simulations of metacommunity interaction networks. They found that the response of communities to restoration depended on how fragmented and damaged the landscape was prior to restoration, with highly damaged and fragmented landscapes being more difficult to achieve community recovery (Gawecka and Bascompte, 2023). Overall, restoration efficiency was increased using a non‐random strategy, choosing patches of habitat to restore that were adjacent to patches with species rich communities (Gawecka and Bascompte, 2023). This suggests that, with high costs of restoration and limited resources available, restoration efforts need to be focused on areas proximal to intact communities to increase the likelihood of success.

Simler‐Williamson and Germino (2022) also investigated non‐random treatment applications in ecological restoration of sagebrush steppe post‐wildfire. They found that post‐fire restoration treatments were disproportionately applied to *more* degraded sites, leading to the conclusion that most restoration efforts are unsuccessful (Simler‐Williamson and Germino, 2022). Thus, the authors advocate for consideration of non‐random treatment strategies to assist in the development of theoretical frameworks for restoration (Simler‐Williamson and Germino, 2022). We interpret these findings as complementary to those of Gawecka and Bascompte (2023), where restoration success of wastelands in fragmented landscapes may be enhanced by choosing sites adjacent to high‐quality habitats.

To assist restoration practitioners, we propose a theoretical framework for assessing restoration priorities and management methods for anthropogenic wastelands based on the degree of fragmentation and proximity to high‐quality habitats within landscapes (Figure 1). We define high‐quality habitat as areas that can provide many propagules of target restoration species, with minimal propagule pressure from undesirable species. Explicit consideration of restoration site position at both the local and the landscape scale may give practitioners a greater chance of restoration success, optimizing funding use and increasing habitat connectivity over time. We recognize that frameworks for restoration that include broader spatial planning are becoming more common (Doherty et al., 2022; Simler‐Williamson and Germino, 2022; Gawecka and Bascompte, 2023) and are not necessarily unique to prioritization of anthropogenic wastelands. However, our focus on anthropogenic wastelands in particular stems from a general lack of information on “where to start” when attempting restoration of these areas.

**Figure 1:**
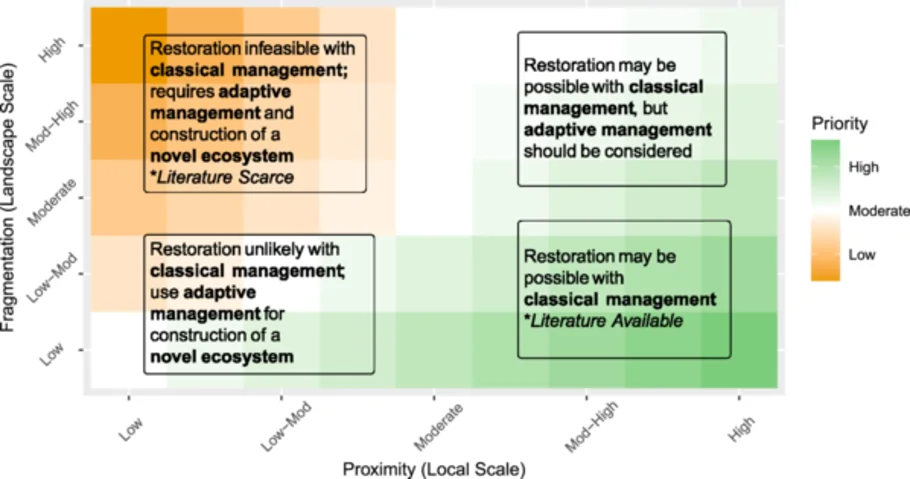
An anthropogenic wasteland restoration framework depicted as a heatmap for assessing priorities and management strategies for wastelands in local proximity to high‐quality habitats from low to high (x‐axis), and landscapes that are unfragmented (low fragmentation) to highly fragmented (y‐axis). Highest priority sites are shown in green, and transition to orange for lowest priority sites for attempted restoration. Text overlays show the recommended restoration strategies for different priority sites. Classical management refers to techniques that aim to restore “historic” conditions of a site and often use singular management methods such as revegetation with native species to achieve management goals. Adaptive management refers to techniques that use multiple methods to meet multiple goals capable of multiple functional endpoints. A novel ecosystem is a unique assemblage of organisms and site characteristics that may be assembled to meet restoration goals.

This framework is intended as a starting point to explore wasteland restoration site priority, as well as which management methods may be most beneficial. Thus, we suggest a range of management approaches for high to low‐priority restoration sites. We define a fragmented landscape as consisting of many isolated patches of habitat, separated by land use such as human development or agriculture. We define proximity to high‐quality habitats as the inverse of distance from these areas (1/distance). Across these scenarios, we identify management strategies (e.g., classical or adaptive) that are likely to achieve successful restoration. We note that while classical management approaches may be effective in the highest priority sites, adaptive management strategies and creation of novel or hybrid ecosystems during restoration are far more likely to achieve restoration goals, especially in moderate to low‐priority sites. When applying this framework (Figure 2), areas proximal to high‐quality habitats should be prioritized over sites further away from these propagule sources, and classical management may be successful in landscapes with little fragmentation (Figure 2A), while adaptive management strategies are recommended to achieve successful restoration in fragmented landscapes (Figure 2B). For instance, restoration of roadside wastelands (characterized by heavy chemical inputs, mowing, invasive species dominance) in fragmented landscapes may be enhanced by selecting sites adjacent to high‐quality habitats and using adaptive management strategies.

**Figure 2:**
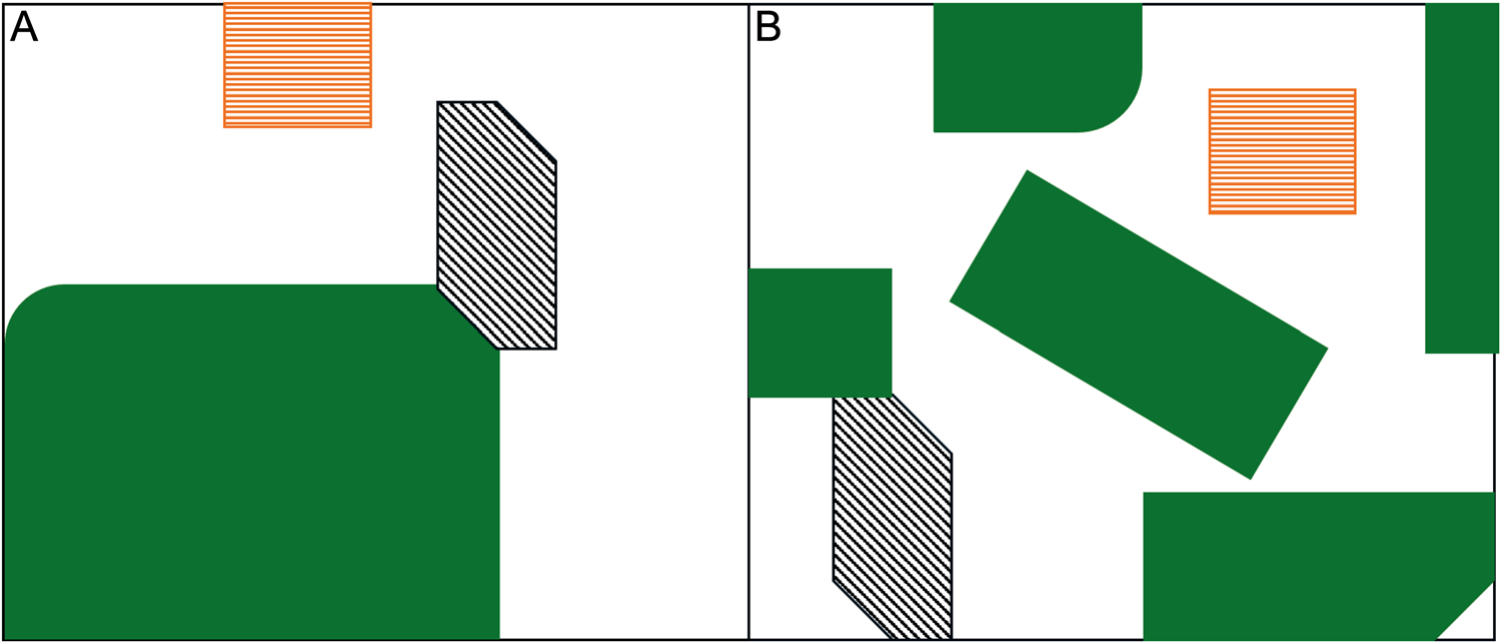
Representation of landscape fragmentation and proximity to natural areas (1/distance), where dark‐green patches represent high‐quality habitats, orange hatched squares are restoration areas in low proximity to high‐quality natural areas, and black hatched polygons are restoration areas in high proximity to high‐quality natural areas. (A) Landscape area with low fragmentation, where classical management strategies may be successful for the high priority, proximal restoration area, but adaptive management strategies should be used for the medium‐priority, distal restoration area. (B) Landscape area with high fragmentation, where adaptive management strategies are recommended for both restoration areas, and restoration of the proximal area is of higher priority than the area further away from high‐quality habitats.

Although this framework may provide practitioners with a place to start, there are questions that remain unanswered. With little known about methods that are effective for vegetation‐focused restoration of anthropogenic wastelands, and the costs associated with this type of restoration, the field would benefit from targeted research and field‐based experimentation to determine the best practices for enhancing success under such challenging conditions. Using this framework, can we lower management inputs and increase successful outcomes by prioritizing sites? It is important to note that highly fragmented landscapes with sites considered wastelands require far more assessment of methodology, effectiveness, and costs associated with restoration, as it is unclear how successful different strategies will be when implemented. Recent literature arguing both for and against spatial prioritization for restoration purposes, regardless of stance, agree that more studies are required to better assess the impact of landscape and restoration site fragmentation overall (Watts and Hughes, 2024; Riva et al., 2025).

Looking forward, it is essential that the limited resources available for restoration be focused on areas with a high likelihood of success (high priority), and it is clear that as the number and scope of areas considered anthropogenic wastelands increases, the need for understanding how to restore these areas grows. Future work necessitates greater reporting on restoration attempts, restoration costs, management strategies, and positioning restoration site choices at multiple scales. While restoration of anthropogenic wastelands is undoubtedly a difficult undertaking, addressing restoration in this context is still worthwhile and will become more successful with focused efforts and greater knowledge of restoration strategies.

## AUTHOR CONTRIBUTIONS

E.S. was responsible for conceptualization and writing (original draft). K.G.T. and J.B.G. were responsible for conceptualization and writing (review and editing).

## Data Availability

Data sharing is not applicable, no data were used in the creation of this article.
